# Whole exome sequencing and deep sequencing of esophageal squamous cell carcinoma and adenocarcinoma in Japanese patients using the Japanese version of the Genome Atlas, JCGA

**DOI:** 10.1007/s10388-021-00835-z

**Published:** 2021-04-07

**Authors:** Eisuke Booka, Yasuhiro Tsubosa, Tomoya Yokota, Shuhei Mayanagi, Kenjiro Ishii, Kenichi Urakami, Keiichi Ohshima, Shumpei Ohnami, Takeshi Nagashima, Ken Yamaguchi

**Affiliations:** 1 Division of Esophageal Surgery, Shizuoka Cancer Center Hospital, 1007 Shimonagakubo, Nagaizumi-cho, Sunto-gun, Shizuoka, 411-8777 Japan; 2 Division of Gastrointestinal Oncology, Shizuoka Cancer Center Hospital, Shizuoka, Japan; 3 Cancer Diagnostic Research Division, Shizuoka Cancer Center Research Institute, Shizuoka, Japan; 4 Medical Genetics Division, Shizuoka Cancer Center Research Institute, Shizuoka, Japan; 5 SRL, Tokyo, Japan; 6 Shizuoka Cancer Center, Shizuoka, Japan

**Keywords:** Esophageal cancer, Esophageal squamous cell carcinoma, Esophageal adenocarcinoma, Mutational signature, Whole exome sequencing, Deep sequencing

## Abstract

**Background:**

Recent comprehensive mutation analyses have revealed a relatively small number of driver mutations in esophageal cancer, implicating a limited number of molecular targets, most of which are also implicated in squamous cell carcinoma.

**Methods:**

In this study, we investigated genetic alterations in 44 esophageal squamous cell carcinomas (ESCC) and 8 adenocarcinomas (EAC) from Japanese patients as potential molecular targets, based on data from the Japanese version of The Genome Atlas (JCGA).

**Results:**

Esophageal cancer was characterized by *TP53* somatic mutations in ESCC (39/44, 88.6%) and EAC (5/8, 62.5%). In addition to *TP53* mutations, somatic mutations in *NFE2L2* (16/44, 36.4%), *CDKN2A* (7/44, 15.9%), and *KMT2D* (7/44, 15.9%) were more frequently detected in ESCC than in EAC. *WRN*-truncated type mutations that lead to genomic instability correlate with EAC, but not ESCC. ESCC samples were enriched in *ALDH2*-associated mutational signature 16 as well as the APOBEC signature. Patients with *FAT2* mutations had significantly poorer overall survival compared with those with wild-type status at *FAT2* (*p* < 0.05). Patients with *EP300* or *PTPRD* mutations also had poor progression-free survival compared with respective wild-types (*p* < 0.05 or *p* < 0.001).

**Conclusions:**

These findings may facilitate future precision medicine approaches based on genomic profiling in ESCC and EAC.

## Introduction

Esophageal cancer is the sixth leading cause of cancer-related mortality worldwide because of its high malignant potential and poor prognosis [1]. The postoperative five-year survival rate in patients with American Joint Committee on Cancer stage I esophageal cancer is approximately 90%; this rate decreases to 45% in patients with stage II disease, 20% in stage III disease, and 10% in stage IV disease [2].

The incidence and histologic subtypes of esophageal cancer exhibit considerable geographic variation [3]. Therefore, esophageal squamous cell carcinoma (ESCC) and esophageal adenocarcinoma (EAC) are good candidates for the analysis of genetic factors that may contribute to the differences in incidence Overall, ESCC is the most frequent esophageal cancer subtype internationally and predominates in eastern Asia and parts of Africa. Tobacco and alcohol consumption are the major risk factors for ESCCs, but other environmental influences including nitrosamines, nutritional deficiencies, specific carcinogens, low socioeconomic status, limited intake of fruits and vegetables, and consumption of very hot beverages have also been implicated in specific geographic regions [4]. In contrast, EAC is the dominant subtype in Western countries and is one of the most rapidly increasing cancers [5]. Its increasing incidence has been associated with a corresponding increase in gastroesophageal reflux disease (GERD) and obesity [5]. Chronic GERD and its development into Barrett’s esophagus are the major risk factors for EACs, along with tobacco use and obesity [6].

The molecular alterations underlying esophageal carcinogenesis have been studied in some depth [3]. *TP53 *point mutations occur in at least 50% of esophageal cancer cases [7]. *TP53 *mutations have also been detected in early stages of ESCC and EAC tumorigenesis as well as in benign Barrett’s esophagus mucosa [8]. A host of additional genes have been studied for mutations in esophageal cancer, but in most of these single-gene studies, very few mutations were identified [3]. To our knowledge, a comprehensive evaluation of all coding regions for mutations has not yet been undertaken in esophageal cancer; thus, it is not yet known whether any previously unstudied genes are commonly mutated in these tumors. Furthermore, it has not been determined whether or not the mutational spectra of ESCCs and EACs differ.

In our previous study, we evaluated 5521 fresh frozen tumor tissues obtained from 5143 Japanese cancer patients through whole exome sequencing (WES), cancer gene panel sequencing, fusion gene panel sequencing and microarray-based gene expression profiling (GEP), thereby establishing the Japanese version of The Cancer Genome Atlas (JCGA) [9]. In our previous study, we reported analyses that summarized all cancers, however, we did not report the specifics of each cancers. In the present study, we focus on esophageal cancer by evaluating 44 patients with ESCCs and 8 patients with EACs from JCGA, and conducted a comprehensive study of esophageal cancer exomes, comparing the two principal histologic subtypes, EACs and ESCCs.

## Materials and methods

### Ethical statement

All experimental protocols were approved by the Institutional Review Board of the Shizuoka Cancer Center (Authorization number 25–33). Written informed consent was obtained from all patients participating in this study. All experiments using clinical samples were performed in accordance with approved Japanese ethical guidelines (human genome/gene analysis research, 2017, provided by Ministry of Health, Labor, and Welfare; https://www.mhlw.go.jp/stf/seisakunitsuite/bunya/hokabunya/kenkyujigyou/i-kenkyu/index.html).

### Subjects

Our present study is a detailed investigation of esophageal oncogene mutations in JCGA. Between January 2014 and March 2019, the samples were obtained from 52 patients with esophageal cancer (44 ESCC and 8 EAC) undergoing surgery at the Shizuoka Cancer Center Hospital, Shizuoka, Japan. We performed WES and deep sequencing of the custom cancer panel (CCP) using blood samples collected during the surgery and fresh surgical specimens after surgery. We then conducted GEP using matched tumor and adjacent normal tissues from each patient. We used only surgical specimens and not biopsy specimens. Therefore, superficial cancers and lesions that were reduced by preoperative chemotherapy were excluded due to the lack of sufficient specimens. The tumor samples were visually assessed by a clinical pathologist in our hospital when tumor content was ≥ 50%, and they were not further filtered by pathophysiological features or cancer type.

WES/CCP and GEP were performed using the Ion Proton system and Agilent system, respectively. Details of the experimental procedures have been described in previous reports [9, 10]. The mean depth of coverage of the target regions was 148.1-fold for WES and 1,135.1-fold for CCP.

### Statistical analysis

Statistical analyses were performed using SPSS version 27.0 software (IBM Corp., Armonk, NY, USA). Categorical data were analyzed using the chi-squared test. Survival was analyzed using the Kaplan–Meier method and log-rank test. *P* < 0.05 was considered significant. For t-Distributed Stochastic Neighbor Embedding (t-SNE) analysis, we performed analyses in the “Rtsne” package (https://github.com/jkrijthe/Rtsne) using our GEP dataset from JCGA [9]. The chi-squared test was applied to compare the genotype between wild-type homozygous and heterozygous or mutated homozygous of *ADH1B* and *ALDH2*.

## Results

### WES and CCP in ESCC and EAC

In the 52 esophageal cancer cohort, all 44 ESCC and 6 of 8 EAC cases were located in the thoracic esophagus, while 2 of 8 EAC cases were located in the esophago-gastric junction (EGJ). The clinical characteristics are shown in Table 1. ALDH2 deficiency was significantly higher in ESCC patients than EAC patients (*P* = 0.039). No relationship with ADH1B genotype was observed for ESCC and EAC. Almost all patients in both ESCC (37/44, 84.1%) and EAC (6/8, 75.0%) were cStage III as defined by the eighth edition of the Union for International Cancer Control TNM classification scheme [11]. Most ESCC patients received preoperative chemotherapy (36/44, 81.8%), while most EAC patients underwent surgery without preoperative chemotherapy (6/8, 75.0%) (*P* = 0.002).

**Table 1:** Characteristics of the esophageal cancer patients

Characteristic	Adenocarcinoma	Squamous cell carcinoma	*p* value
Total number	8	44	
Age (years)			0.646
≦50	0	2	
51–60	2	5	
61–70	3	23	
≧71	3	14	
Gender			0.267
Male	8	38	
Female	0	6	
Smoking status			0.764
Nonsmokers	1	4	
Smokers	7	40	
Pack-years^a^			0.677
0	1	4	
Light smokers (> 0 to < 20)	2	6	
Heavy smokers (≥ 20)	5	33	
Smokers but pack-years unknown	0	1	
Drinking status			0.585
Nondrinkers	0	2	
Drinkers	6	40	
Unknown	2	2	
Genotype^b^			
ADH1B			0.677
His/His	5	24	
His/Arg	3	14	
Arg/Arg	0	6	
ALDH2			0.039
Glu/Glu	4	9	
Glu/Lys	3	34	
Lys/Lys	0	1	
U.D.^c^	1	0	
cStage (UICC TNM 8th)			0.805
I	0	0	
II	1	4	
III	6	37	
IV	1	3	
Neoadjuvant therapy			0.002
Chemotherapy	2	36	
Chemoradiotherapy	0	1	
None	6	7	
Surgical procedure			< 0.001
Subtotal esophagectomy	4	39	
Lower esophagectomy	4	1	
Pharyngolaryngectomy with esophagectomy	0	4	
pStage (UICC TNM 8th)			0.083
I	0	1	
II	0	9	
III	4	28	
IV	4	6	

^a^Pack-years defined as number of packs of cigarettes smoked per day times of years of smoking

^b^Genotype defined as AA vs Aa + aa for dominant model. A and a are the major and minor alleles, respectively

^c^U.D.; undetectable sequence by insufficient depth

ADH1B; His47Arg^d^ (rs1229984)

ALDH2; Glu487Lys^d^ (rs671)

^d^Amino acid position based on Refseq NM_000668 (ADH1B) and NM_000690 (ALDH2), respectively

**Fig. 1:**
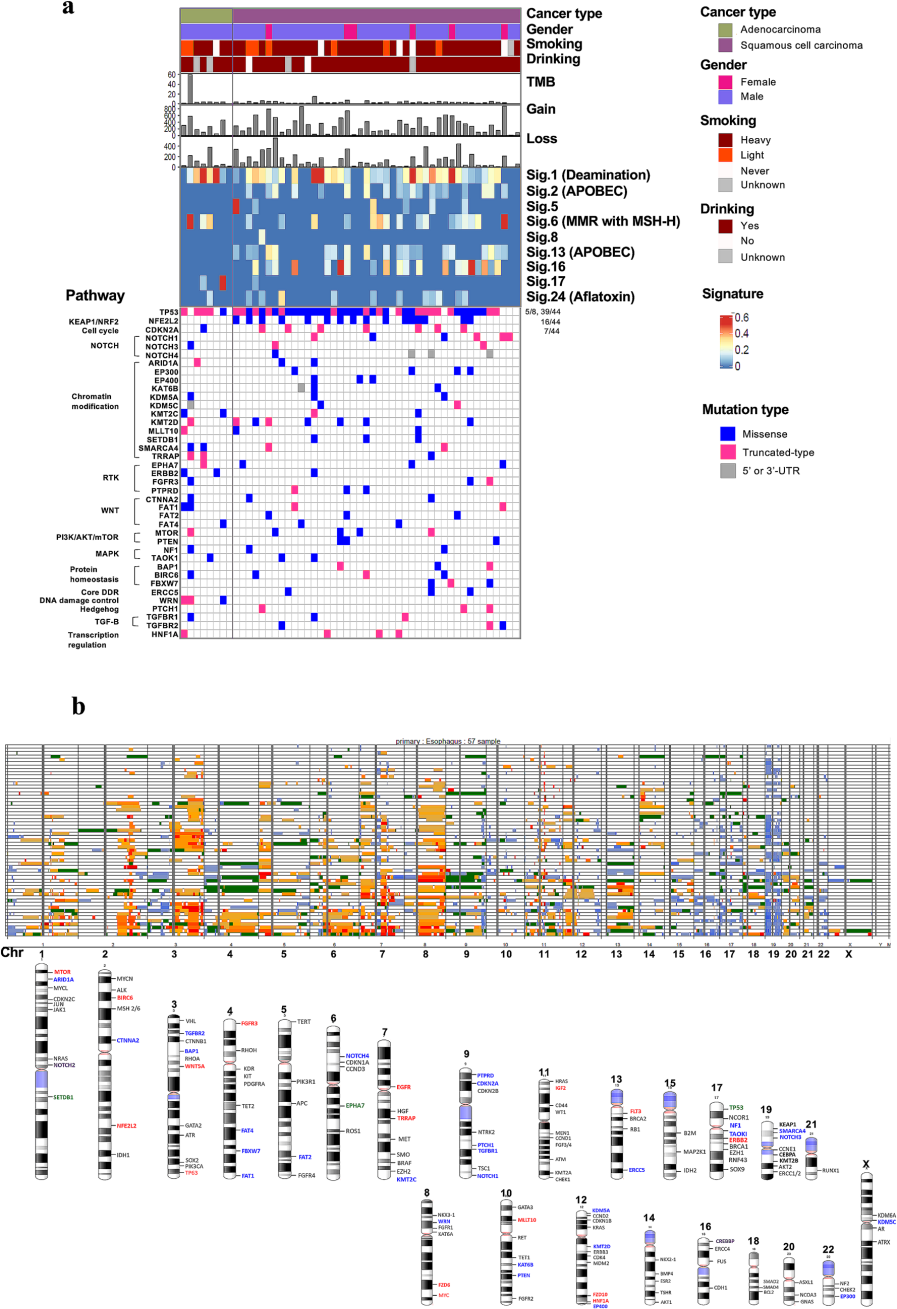
**a** Mutation signatures, and pathway alterations are shown. Cancer type, gender, smoking history, drinking history, tumor mutation burden (TMB), gain, loss, signature contribution, and pathway contribution are shown from the top to bottom, respectively. Each row represents a sample. **b** Copy number alterations. Figure shows amplifications in red and deletions in green for chromosomes 1 to 20 and X

*UICC* International Union against Cancer, *TNM* tumor, nodes, and metastasis,

We used WES to analyze 1,074 cancer-related genes from 27 databases in paired tumor tissue and blood samples to detect genetic differences between ESCC and EAC [9]. Simultaneously, we used a CCP that 409 target genes to conduct deep sequencing of tumor tissue samples to validate the WES data. We focused on genes that are classified as tumor suppressor genes (TSG) or oncogenes with 3 or more mutations (Fig. 1a). *TP53* mutations were detected in 88.6% (39/44) of ESCC and 62.5% (5/8) of EAC samples, which was consistent with the observed frequencies for these mutations from a previous study (93.1% in ESCC and 72% in EAC) [12, 13]. Among ESCC samples, somatic mutations in *NFE2L2* were the second most frequently detected (16/44, 36.4%) after *TP53* mutations, but no *NFE2L2* mutations were detected in EAC cases. Somatic mutations in *CDKN2A* (7/44, 15.9%), *KMT2D* (7/44, 15.9%) were frequently detected in ESCC compared to EAC. EAC is related to *WRN*-truncated type mutations that lead to genomic instability in cancers, but not ESCC. Various pathways were observed to be enriched in ESCC, including those related to *KEAP1*/*NRF2* signaling, cell cycle, *NOTCH* signaling and chromatin modification. Moreover, ESCC samples were characterized as being predominantly attributed to mutational signatures 2 and 13, associated with the APOBEC family, and signature 16, which has a high contribution rate in *ALDH2* mutation that is related to alcohol metabolism (Fig. 1a) [14]. Next, copy number aberrations were analyzed for ESCC samples. We observed predominantly copy number gains in 2q, 3q, and 8q, while copy number losses were observed in 17p and 19p (Fig. 1b).

**Fig. 2:**
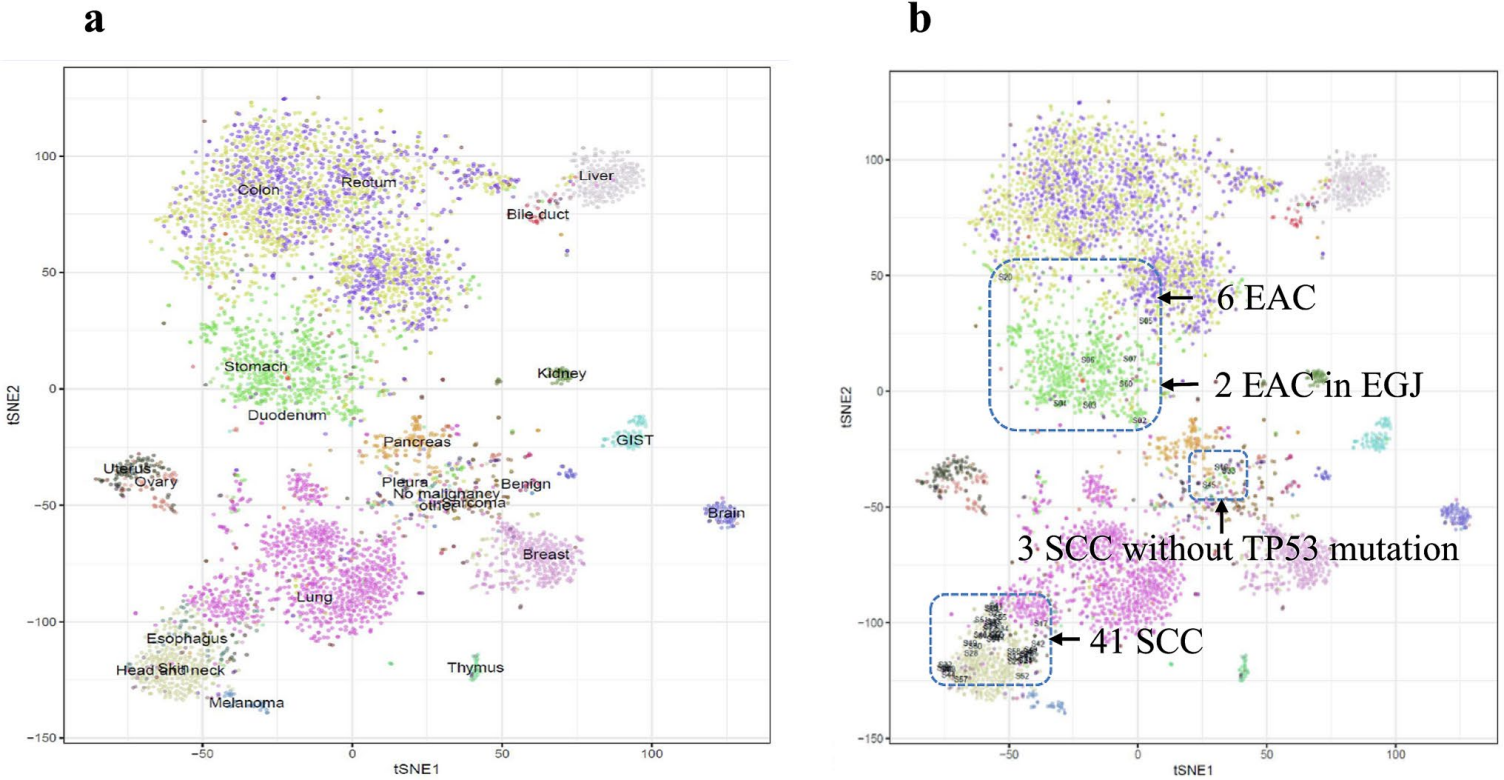
**a** A t-Distributed Stochastic Neighbor Embedding (t-SNE) analysis was performed based on total gene expression data from 5143 patients. **b** 44 ESCCs and 8 EACs were plotted and are circled with a blue dotted line

A two-dimensional t-SNE analysis using the “Rtsne” package, based on comprehensive GEP data in various tumors were shown in Fig. 2a. The t-SNE plots were distributed along with the expression levels of individual cancer types, in which all 8 EAC patients including the 2 EGJ cases showed an analogous expression pattern to adenocarcinoma of stomach and duodenum. Among the 44 ESCC, 41 ESCC cases showed analogous expression patterns to squamous cell carcinoma of the skin, head, and neck, and 39 of these 41 ESCC had *TP53* mutations. It is noteworthy that all of the three cases of ESCC that were illustrated at positions different from squamous cell carcinoma of esophageal cancers were cases without *TP53* mutation (Fig. 2b).

### Effect of genetic alterations on survivals

**Fig. 3:**
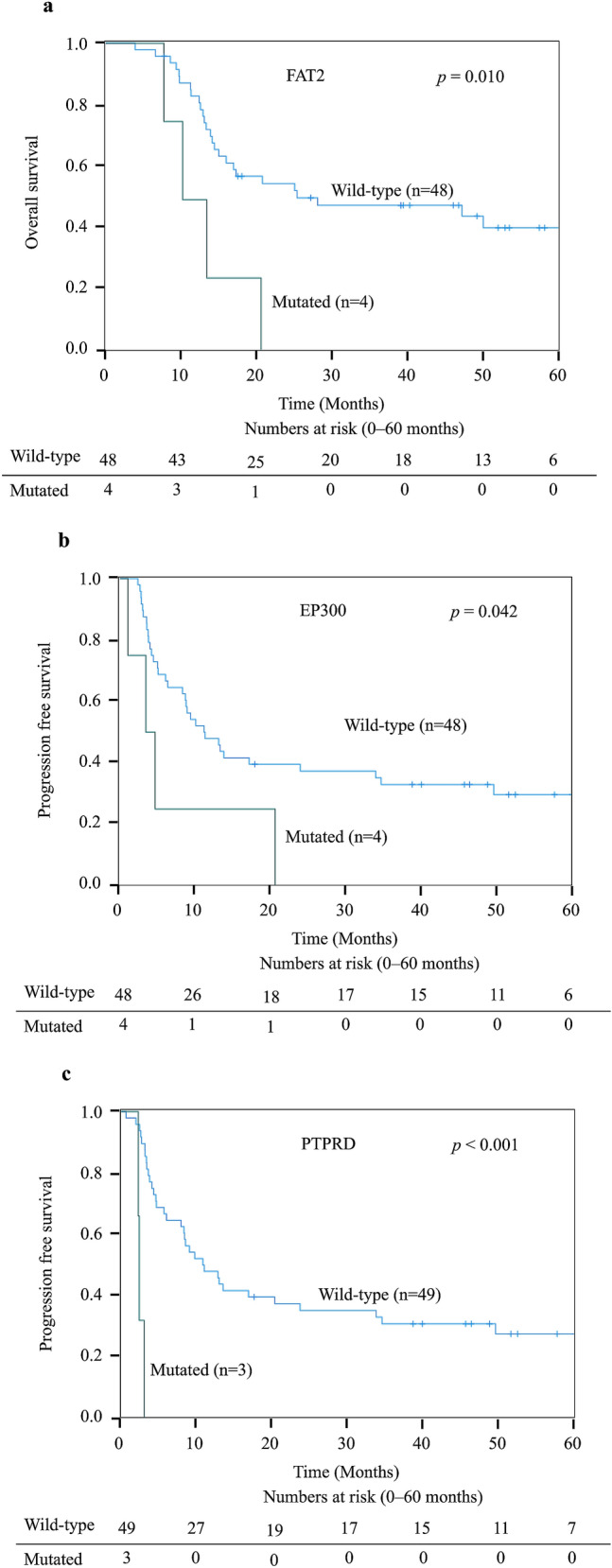
Kaplan–Meier survival curves for patients with or without mutated genes: **a** overall survival compared FAT2 mutated and wildtype, **b** progression-free survival compared EP300 mutated and wildtype, and **c** progression-free survival compared PTPRD mutated and wildtype

To assess the clinical impacts for targeted genes that were selected in Fig. 1a, we analyzed the impacts of targeted genes on overall survival (OS) and progression-free survival (PFS) using the Kaplan–Meier method and log-rank test. After analyzing all genes in Figs. 1a, 3 genes had negative impact on survivals significantly. Patients with *FAT2* wildtype had significantly (*p* < 0.05) better OS compared with those in with *FAT2* mutations (Fig. 3a), and patients with *EP300* and *PTPRD* mutations had worse PFS, compared to wildtype, respectively (Fig. 3b, c). Four patients with *FAT2* mutations included 3 pStageIII ESCC and 1 pStageIV EAC. Four patients with *EP300* mutations included 1 pStageI, 2 pStageIII and 1 pStageIV ESCC. Three patients with *PTPRD* mutations included 3 pStageIII ESCC. These 3 genes with negative impact on survivals had no relationship with pathological characteristics.

### Comprehensive gene expression analysis in the EAC and ESCC samples

**Fig. 4:**
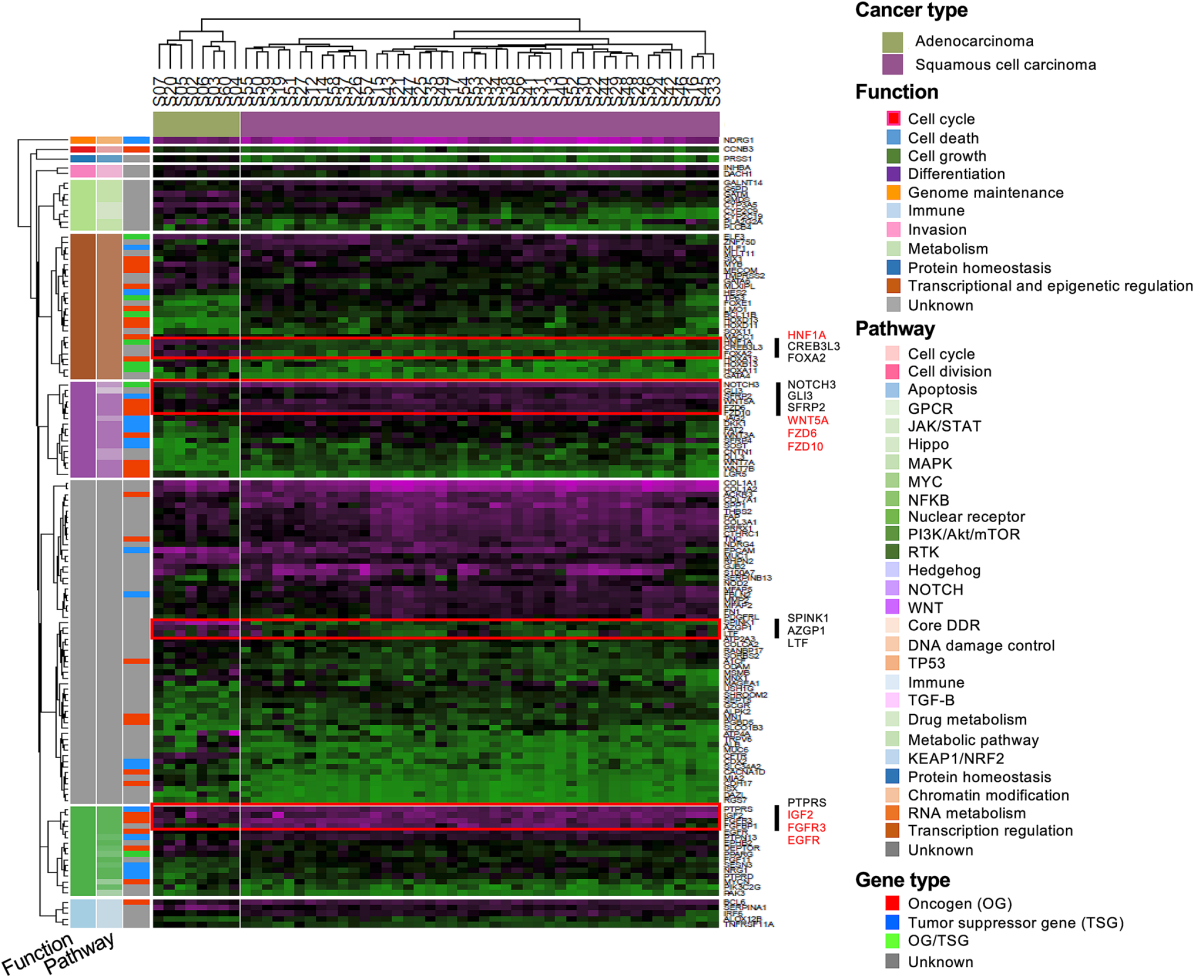
Differences in ESCC and EAC expression patterns were shown for the 1998 cancer-related genes. Genes were clustered by fixing the cancer type and function or pathway. Genes where the expression difference was observed between ESCC and EAC was highlighted in red

Of the genes in a known pathway or function, transcription factors, such as *MECOM*, *TMPRSS2*, *GATA5*, *HNF1A*, *CREB3L3*, and *FOXA2*, were upregulated in EAC. It is noteworthy that unknown pathway genes, *SPINK1*, *AZGP1*, and *LTF* were upregulated in EAC cases, but not in ESCC (Fig. 4). On the other hand, NOTCH pathway (*NOTCH3*), Hedgehog pathway (*GLI3*), WNT pathway (*SFRP2*, *WNT5A*, *FZD6/10*) were upregulated characteristically in ESCC (Fig. 4), which was consistent with the alterations observed in previous study [12].

## Discussion

Risk factors for ESCC include drinking and smoking, however, the International Agency for Research on Cancer (part of the World Health Organization) has identified acetaldehyde, which is associated with alcoholic beverages, as a clear carcinogen in addition to drinking and smoking [15]. Patients with ALDH2 deficiency who cannot decompose acetaldehyde, the primary metabolite of ethanol contained in alcoholic beverages, are at high risk of esophageal cancer if they drink habitually [16]. The frequency of ALDH2 deficiency is less than 1% in Caucasians and about 50% in Japanese populations [16]. In this study, 35 of 44 ESCC patients (79.5%) were ALDH2 deficiency, on the other hand 3 of 7 EAC patients (42.9%) are ALDH2 deficiency. The frequency of ALDH2 deficient mutations in EAC seemed to be equivalent to the frequency in Japanese populations. In the case of ESCC, the frequency of ALDH2 deficiency was increased compared to the Japanese population, indicating that ALDH2 deficiency may be specifically associated with ESCC as found in a previous report [16].

Our study is the first multi-omics analysis from a single-institution that can be matched to clinical data. In the current study, we performed multi-omics analysis in 52 esophageal cancer patients compared with 5143 other types of cancer patients, and we firstly reported the genomic location of EAC and ESCC in the JCGA [9]. EAC showed analogous expression patterns to adenocarcinoma of the stomach and duodenum, and ESCC showed analogous expression patterns to squamous cell carcinoma of the skin, head, and neck. Notably, the three cases of ESCC plotted at positions different from other 41 ESCC were all cases without TP53 mutation. Moreover, among the three cases of ESCC without TP53 mutation, 2 patients had no history of smoking and were found to have a NOTCH1 mutation. From the results, it is possible that TP53 mutations are strongly associated with squamous cell carcinoma of skin, and head and neck as well as ESCC, and ESCC without TP53 mutations are genetically distinct from ESCC with TP53 mutations. It is also possible that ESCCs without TP53 mutation were correlated with NOTCH1 mutation without history of smoking.

Recently, two studies have reported that the frequent mutations in normal esophageal tissues were TP53 and NOTCH1 [4, 17]. On the other hand, in the examination of ESCC, TP53 mutations were overwhelmingly predominant in both reports, and NOTCH1 mutations were less frequent than in normal tissues [4, 17]. In our study, there were three ESCC cases plotted at positions different from the other 41 cases of ESCC. These three cases were all without TP53 mutations, and two of the three cases were with NOTCH1 mutations. Moreover, these two patients with NOTCH1 mutation had no history of smoking. It is possible that carcinogenesis is accelerated by adding the risk of drinking and smoking to the TP53 mutation, however NOTCH1 mutation is associated with carcinogenesis regardless of drinking and smoking.

Accentuated in liver cancers from Japanese men, signature 16 has recently been related to alcohol consumption among Asian patients with ESCC, on the basis of its association with alcohol drinking and two risk alleles for ESCC that are involved in alcohol metabolism (ALDH2 (rs67) and ADH1B (rs122998)) [18]. The association with ALDH2 risk allele was also confirmed in esophagus, hepatic and stomach cancers from The Cancer Genome Atlas (TCGA) [4]. In our study, APOBEC (signature 2 and 13) and signature 16 were highly enriched in mutations in ESCCs, however were not seen in EACs at all. Yokoyama et al. reported that APOBEC signature was predominant in ESCC which is consistent with our study [4]. Recently, signature 16 was reported to be associated with gastric cancer of alcohol consumers with an ALDH2 defective allele (rs671) [14]. Signature 16 was highly enriched with ALDH2 deficiency in ESCCs in our study. This suggests that signature 16 could be associated with alcohol consumption in carcinogenesis irrespective of the specific cancer type.

The genomic difference between ESCC and EAC based on microarray could lead to further guidelines for treatment especially for chemotherapy. At the moment, EAC is either treated as esophageal cancer or gastric cancer depending on the institute. The results of this study suggested that EACs were genetically more similar to gastric cancer and therefore could be treated in the same way as gastric cancer. EAC is considered to be similar to gastric cancer, especially in terms of chemotherapy susceptibility. Esophageal cancer is mainly treated with cisplatin and 5-FU, whereas gastric cancer is mainly treated with S-1 and platinating agent [19–21]. Accordingly, EAC might have better outcomes when treated with S-1 and platinating agent than cisplatin and 5-FU and the effect of chemotherapy on EAC should therefore be assessed in future studies.

Unlike previous reports, deep sequencing was performed for esophageal cancer for the first time [12, 13, 22–27]. As in previous reports, a high frequency of mutations were found in TP53, NFE2L2, and CDKN2A genes in esophageal cancer based on our deep sequencing results and suggest that these gene mutations could be established as contributors to esophageal cancer tumorigenesis [12, 13, 22, 23]. In this study, differences in the impact of TP53 and NOTCH1 mutations on esophageal cancer carcinogenesis were implied. Further examination of these differences could lead to the discovery of drug-related driver mutations. In addition, unknown pathway genes, *SPINK1*, *AZGP1*, and *LTF* were upregulated in EAC cases, but not in ESCC in our deep sequencing. Further examination of these genes could reveal possible molecular pathways for these genes and may be warranted in understanding EAC.

To date, there is not much genetic analysis of esophageal cancer that can be matched with clinical data, so the association of genetic alterations with patient prognosis and mutational signatures are unclear. EP300 mutation was a significant poor prognostic factor in this study, which is consistent with previous reports [12, 22]. EP300 mutations are a promising candidate for significant poor prognosis. Although no EP300-specific drugs have been discovered as yet, it has been suggested that EP300-specific drug development could improve the prognosis of advanced ESCC in the future. To our knowledge, there have been no reports describing the impact of FAT2 and PTPRD on patient survival. In this study, we report the negative impact of FAT2 and PTPRD mutations on long-term survival and these candidates warrant further investigation. We believe that the new therapies targeting EP300, FAT2 and PTPRD mutations will be available in the future. In this study, circulating tumor cells (CTCs) were not detected, however, it was recently reported that gene alterations could be detected in CTCs from liquid biopsy [28]. Liquid biopsy of CTCs was minimally invasive and could be widely used in the future.

This study has several limitations. First, since surgical specimens were used, superficial cancers and lesions that were reduced by preoperative chemotherapy were excluded, so there is a possibility of selection bias. Second, most patients with ESCC received preoperative chemotherapy, and preoperative chemotherapy may have affected the difference in gene alteration between ESCC and EAC.

This study represents a comprehensive characterization of genomic alterations in ESCC and EAC. This could provide insights into the genetic mechanism behind ESCC and EAC tumorigenesis. It will be important to explore the biological and therapeutic significance of these newly discovered mutated and amplified genes, as this may ultimately lead to the development of effective diagnostic and therapeutic approaches for ESCC and EAC.
